# Longitudinal analysis of ovarian cancer death patterns during a rapid transition period (2005-2020) in Shanghai, China: A population-based study

**DOI:** 10.3389/fonc.2022.1003297

**Published:** 2022-10-03

**Authors:** Xiaopan Li, Mo Zhang, Yichen Chen, Huihui Lv, Yan Du

**Affiliations:** ^1^ Department of Health Management Center, Zhongshan Hospital, Shanghai Medical College of Fudan University, Shanghai, China; ^2^ Clinical Research Unit, Obstetrics and Gynecology Hospital, Fudan University, Shanghai, China; ^3^ Office of Scientific Research and Information Management, Center for Disease Control and Prevention & Pudong Institute of Preventive Medicine, Pudong New Area, Shanghai, China; ^4^ Yueyang Hospital of Integrated Traditional Chinese and Western Medicine, Shanghai University of Traditional Chinese Medicine, Shanghai, China

**Keywords:** ovarian cancer, all cause of death, underlying cause of death, mortality, years of life lost, trend analysis, decomposition method

## Abstract

**Objectives:**

It is important to assess the burden of ovarian cancer related premature death so as to develop appropriate evidence-based care and improve women’s health. This study aimed to characterize the long-term trends in mortality, survival and disease burden of ovarian cancer in Shanghai, China.

**Materials and Methods:**

Co-morbidities, crude mortality rate (CMR), age-standardised mortality rate by Segi’s world standard population (ASMRW), years of life lost (YLL), and survival rates were analysed. Temporal trends for the mortality rates and disease burden were analyzed using the Joinpoint Regression Program. Mortality rate increases by demographic and non-demographic factors were estimated by the decomposition method.

**Results:**

A total of 1088 ovarian cancer as underlying cause of deaths were recorded. CMR and ASMRW were 4.82/10^5^ and 2.32/10^5^ person-years, respectively. The YLL was 16372.96 years, and the YLL rate was 72.46/10^5^ person-years. The YLL rate increased only in the age group of 70-79 years (P = 0.017). The survival rates of ovarian cancer patients did not improve during the ten year period (2005-2015). The top co-morbidities were diseases of the respiratory system, digestive system, and circulatory system. The rates of ovarian cancer deaths caused by non-demographic and demographic factors increased by 21.29% (95%CI: 4.01% to 41.44%, P = 0.018) and 25.23% (95%CI: 14.64% to 36.81%, P < 0.001), respectively.

**Conclusions:**

Population ageing and all cause of death may affect ovarian cancer related deaths in Pudong, Shanghai. The high mortality and the stagnant survival rates suggest the need for more efforts in targeted prevention and treatment of this disease.

## Introduction

Ovarian cancer is a type of lethal gynaecologic tumour, which causes significant health issues in women (1). The majority of ovarian cancer patients are diagnosed at the late stage, with poor prognosis. For decades the overall 5-year survival rate of advanced ovarian cancer patients has improved modestly, and is not optimistic even in high-income countries such as the United States (about 47%) (2). Although there have been major improvements in ovarian cancer therapy, challenges remain in the treatment of this aggressive disease (3). Most of the patients initially are sensitive to chemotherapy; however, many of them will develop drug resistance, leading to recurrence and death (3). Therefore, it is important to understand ovarian cancer related mortality, so as to improve the prevention and treatment strategies, including more accurate screening and diagnostic methods, and effective treatment modalities.

The survival of ovarian cancer is associated with age (4), which is a main demographic factor. As for non-demographic factors, levels of medical technology and health consciousness are two main factors of ovarian cancer mortality (5, 6). To the best of our knowledge, currently there is limited research evaluating the all cause of death of ovarian cancer, which may under-estimate the disease burden. In addition, few studies have quantitatively evaluated the contribution of demographic and non-demographic factors to the burden of ovarian cancer. What is more, previous epidemiological studies using indicators such as incidence, mortality, and survival rate cannot reflect the complete disease burden on public health. Years of life lost (YLL) can quantify the premature mortality and reflect the disease burden on society from a particular cause of death (7).

China is one of the largest low-to-middle income countries (LMICs), and has been working diligently to eradicate poverty. Accompanied with rapid economic development, great changes have happened in China’s population and disease spectrum (8). Shanghai is an international metropolitan city located in the east coast of China, and is a high-level economic, industrial, and cultural centre. Since 1990, the Shanghai Pudong New Area (PNA) has been set as a national economic and technological development zone in China (8). Other cities in China and other LMICs are likely to follow the development process of Shanghai in the future (9). With a mixture of urban, suburban, and rural geographic areas, PNA is a good representative of Shanghai. Studying the characteristics of disease changes in the PNA can provide valuable information for understanding the disease burden and developing relevant strategies in regions experiencing rapid social and economic transition.

In this population-based study, we aimed to analyze the long-term trends in the mortality, overall survival, and disease burden of ovarian cancer in Shanghai PNA of China from 2005 to 2020, and characterize the impacts of demographic and non-demographic factors on ovarian cancer mortality.

## Materials and methods

### Data source

Ovarian cancer related death data of registered PNA permanent residents from 2005 to 2020 were obtained from the Mortality Registry System of Shanghai PNA, which covers all medical institutions of the area, the public security system, and the funeral and cremation system, which ensure the data completeness of the registration system. All causes of death were coded by rigorously trained clinicians, and further verified by local Center for Disease Control and Prevention (CDC) (10). The population data were obtained from the Public Security Bureau and the Statistics Bureau of PNA. Shanghai PNA has a permanent resident population of 3.22 million (11). Information about resident patients diagnosed with ovarian cancer between 2002 and 2016 was obtained from the PNA Cancer Registry, and was used for survival analysis (12). The PNA Cancer Registry System started in the year of 2002, which collected data from reports of hospitals with tumor diagnostic qualification, the medical records obtained from autopsy and community follow-up and further verified by local CDC. Periodic evaluation, data cleaning and compilation are performed to maintain the integrity of the registry system at both the county and provincial levels according to standard guidelines (9).

Ovarian cancer (ICD-10: C56) related death (all causes of death) was classified based on the ovarian cancer being a direct or indirect cause (died of other causes other than ovarian cancer) of death according to the International Classification of Diseases 10th version (ICD-10) (13). The direct causes of death include the underlying cause of death, which is a common indicator in the cause-of-death statistics. The all causes of death were coded by physicians according to the conditions of patients and further verified by the local Centre for Disease Control and Prevention (CDC).

The study protocol followed the 2000 Declaration of Helsinki and was approved by the ethics committee of the Shanghai PNA Centre for Disease Control and Prevention (No. 2016-04-0586).

### Statistical analyses

Crude mortality rates (CMR) was calculated as total number of ovarian cancer deaths each year divided by the corresponding annual average female population in PNA and shown as per 100,000 persons (/10^5^). Age-standardised mortality rates by Segi’s world standard population (ASMRW) were obtained and presented also as per 100,000 persons (/10^5^). YLL was calculated based on the method described by Murray and Lopez and was detailed previously (8).

Age were categorized into the groups of 0-4, 5-14, 15-29, 30-44, 45-59, 60-69, 70-79, and ≥ 80 years. Since there were few ovarian cancer related deaths before the age of 30, trends in age-specific CMR, ASMRW, and YLL rate were calculated for the age groups 0-29, 30-44, 45-59, 60-69, 70-79, and ≥ 80 years. Life table method was applied to calculate the 1- to 5- year overall survival rates from 2005 to 2015 (12).

Joinpoint Regression Program 4.3.1.0 (National Cancer Institute, Bethesda, MD, USA) was utilized to calculate the temporal trends of CMR, ASMRW, YLL rate, and survival rate, which were expressed as average annual percent change (AAPC) with corresponding 95% confidence interval (95% CI). Statistic difference of AAPC was assessed using the Z test, with the terms “increase” or “decrease” describing a statistically significant (P < 0.05) AAPC, and “stable” for non-significant trends (8). The increased mortality rates of each year from 2006 to 2020, compared with the data of the first year (2005), related to demographic and non-demographic factors were estimated by the decomposition method, in which mortality rates were calculated and compared for each 5-year age group, from 0–4 to 85+ years (14).

**Figure 1:**
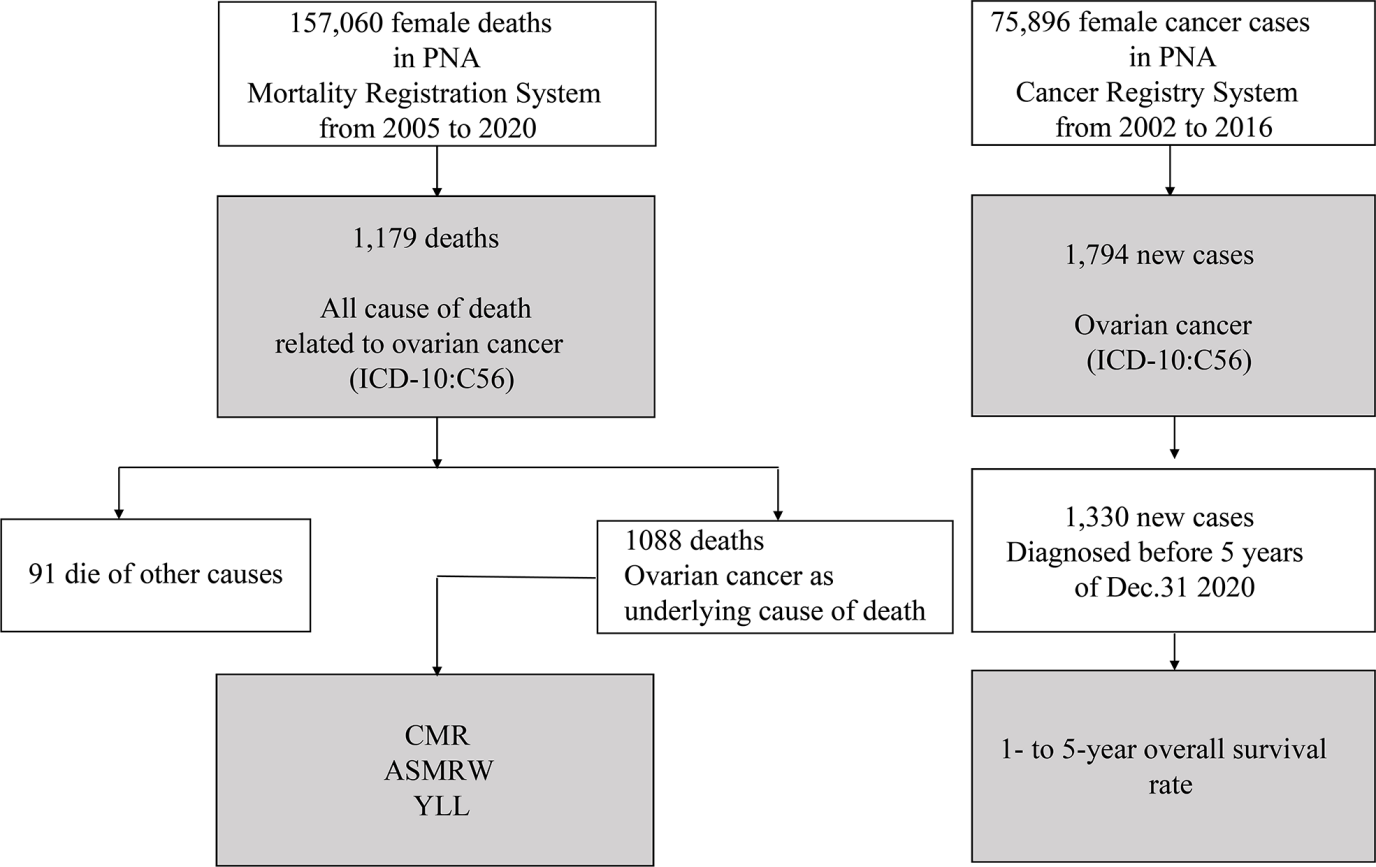
Flowchart of data acquisitions and statistical analyses.

Data acquisitions and process of statistical analysis are presented in **Figure 1**. All statistical analyses were performed using SPSS (version 21.0; SPSS, Inc., Chicago, IL) and R (version 3.4.3). A two-sided P value of < 0.05 was considered as statistically significant.

## Results

### Baseline characteristics of ovarian cancer related deaths

There are a total of 22,595,080 person-years of registered permanent female residents from 2005 to 2020 in PNA. There were a total of 1179 all cause of death related to ovarian cancer (ICD-10: C56) (**Figure 1**), including 1088 (1088/1179, 92.28%) underlying cause of ovarian cancer, and 91 (91/1179, 7.72%) died of other causes (**Supplementary Table S1**). **Table 1** presents the baseline characteristics of ovarian cancer deaths. Of those 1088 underlying cause of death, 1067 (1067/1088, 98.07%) had ovarian cancer as underlying cause of death, and 21 (21/1088, 1.93%) had ovarian cancer and other cancer types as underlying causes of death. The median (mean) age at death was 63.91 years (64.42 ± 13.22 years). CMR and ASMRW were 4.82/10^5^ and 2.32/10^5^ person-years, respectively (**Supplementary Table S2**).

**Table 1:** Baseline characteristics of ovarian cancer death in Pudong, Shanghai, China from 2005 to 2020.

Characteristic	Deaths (n, %)	Age at years (Mean ± SD)	Age at years (Median, range)	CMR (/10^5^)	ASMRW (/10^5^)	YLL (years)	YLL rate (/10^5^)
Total	1088 (100.00)	64.42 ± 13.22	63.91 (20.25-99.03)	4.82	2.32	16372.96	72.46
Periods							
2005-2008	200 (18.38)	62.38 ± 13.72	59.01 (21.24-88.76)	3.85	1.99	3167.79	60.93
2019-2012	255 (23.44)	63.21 ± 13.65	61.52 (21.97-99.03)	4.62	2.35	3969.98	71.95
2013-2016	302 (27.76)	64.58 ± 12.34	63.93 (20.25-91.59)	5.23	2.47	4535.75	78.50
2017-2020	331 (30.42)	66.42 ± 13.13	66.20 (23.87-98.25)	5.43	2.38	4699.43	77.04
Underlying cause of death ovarian cancer							
Underlying cause of death (C56)	1067 (98.07)	64.42 ± 13.27	63.85 (20.25-99.03)	4.72	2.27	16053.68	71.05
Underlying cause of death (C97, including C56 plus other cancers)	21 (1.93)	64.47 ± 10.67	64.46 (44.87-87.27)	0.09	0.05	319.28	1.41
Top three co-morbidities in all causes of ovarian cancer death							
Diseases of the respiratory system (J00-J99)	110 (10.11)	66.46 ± 14.40	67.39 (20.25-94.02)	0.49	0.22	1537.81	6.81
Diseases of the digestive system (K00-K93)	54 (4.96)	60.43 ± 10.64	59.33 (33.75-83.87)	0.24	0.12	914.61	4.05
Diseases of the circulatory system (I00-I99)	48 (4.41)	65.21 ± 13.92	65.05 (35.88-90.35)	0.21	0.10	706.29	3.13
Metastatic carcinoma (C00-C96)	336 (30.88)	61.48 ± 12.02	60.73 (21.97-91.59)	1.49	0.77	5509.36	24.38
Metastatic ovarian cancer to the liver (C78.7)	51 (4.69)	60.42 ± 9.86	60.01 (37.52-85.54)	0.23	0.12	862.69	3.82
Metastatic ovarian cancer to the lung (C78.0)	27 (2.48)	64.13 ± 11.07	60.50 (37.52-84.18)	0.12	0.05	415.80	1.84
Metastatic ovarian cancer to the retroperitoneum and peritoneum (C78.6)	48 (4.41)	66.77 ± 11.17	64.36 (44.13-88.38)	0.21	0.10	681.52	3.02
Metastatic ovarian cancer to unspecified sites (C79)	187 (17.19)	60.36 ± 12.39	60.01 (21.97-91.59)	0.83	0.45	3139.61	13.90

ASMRW, age-standardised mortality rate by Segi’s world standard population; CMR, crude mortality rate; YLL, years of life lost.

### Main ovarian cancer co-morbidities and metastatic cancer

The main co-morbidities of the death cases were analyzed, and the top three co-morbidities in all causes of ovarian cancer death were diseases of the respiratory system (ICD-10: J00-J99), diseases of the digestive system (ICD-10: K00-K93), and diseases of the circulatory system (ICD-10: I00-I99), with 110 (10.11%), 54 (4.96%), and 48 (4.41%) deaths, respectively. A total of 336 deaths had metastatic carcinoma, accounting for 30.88% of the total deaths. The breaking down of the metastatic carcinoma is shown in **Table 1**. The median/mean age, CMR, ASMRW, and YLL rates of ovarian cancer related deaths of each subgroup are shown in **Table 1** and **Supplementary Tables S2**-**S3**.

### Age-specific ovarian cancer mortality

The proportions of ovarian cancer death of each age category are presented in **Supplementary Tables S4**. The number of deaths from ovarian cancer < 30 years of age was 11 (all in the age group of 15-29), accounting for 1.01% of total deaths; while most of the deaths (n = 364, 33.46%) occurred at the age group of 45-59 years. CMR in the age groups of 0-4, 5-14, 15-29, 30-44, 45-59, 60-69, 70-79, and ≥ 80 years were 0.00, 0.00, 0.31, 1.06, 6.29, 9.26, 12.91, and 13.12/10^5^ person-years, respectively (**Table 2** and **Supplementary Tables S2**).

**Table 2:** Number of deaths, CMR, YLL, and YLL rate by age group.

Age group (years)	Deaths (n)	Proportion (%)	CMR (/10^5^)	YLL (years)	YLL rate (/10^5^)
0-4	0	0.00	0.00	0.00	0.00
5-14	0	0.00	0.00	0.00	0.00
15-29	11	1.01	0.31	300.61	8.42
30-44	53	4.87	1.06	1282.32	25.63
45-59	364	33.46	6.29	7238.99	125.15
60-69	287	26.38	9.26	4395.94	141.59
70-79	222	20.40	12.91	2305.43	134.21
≥ 80	151	13.88	13.12	849.67	73.75
Total	1088	100.00	4.82	16372.96	72.46

CMR, crude mortality rate; YLL, years of life lost.

### Burden of premature death from ovarian cancer

The YLL due to premature death from ovarian cancer was 16372.96 years, and the rate of YLL was 72.46/10^5^ person-years (**Supplementary Tables S3**). Regarding the main co-morbidities in all-cause death, YLL and YLL rate due to respiratory system (ICD-10: J00-J99) were the highest (1537.81 years, 6.81/10^5^), followed by digestive system (ICD-10: K00-K93) (914.61 years, 4.05/10^5^), and circulatory system (ICD-10: I00-I99) (706.29 years, 3.13/10^5^; **Table 1**). The top three age groups of YLL were 45-59, 60-69, and 70-79 years, with YLL of 7238.99, 4395.94, and 2305.43 years, respectively. The top three age groups of YLL rates were 60-69, 70-79, and 45-59 years, with YLL rates of 141.59/10^5^, 134.21/10^5^, and 125.15/10^5^, respectively (**Table 2**).

### Trends of mortality and burden of ovarian cancer

**Figure 2:**
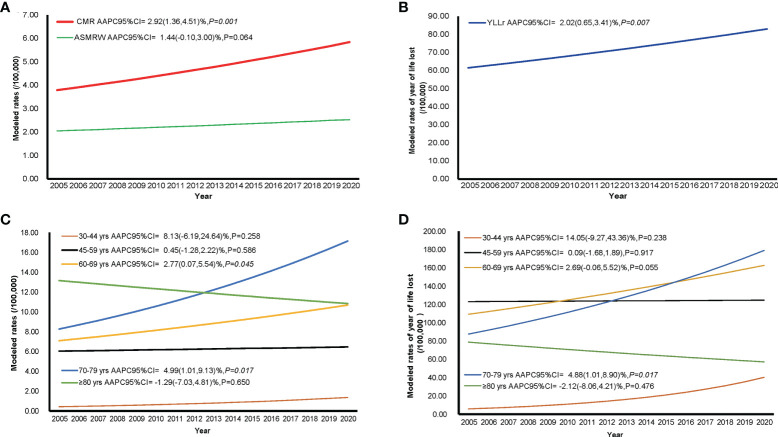
The trends in CMR, ASMRW, and YLL rate of persons with ovarian cancer death in Pudong New Area, Shanghai, China, 2005-2020. **(A)** trends in CMR and ASMRW; **(B)** trends in YLL rate; **(C)** trends in CMR among different age groups; **(D)** trends in YLL rate among different age groups. ASMRW, age-standardised mortality rate by Segi’s world standard population; CMR, crude mortality rate; YLL, years of life lost.

The temporal trends of CMR, ASMRW, and YLL rate were expressed based on the modelled CMR, ASMRW, and YLL rate, as shown in **Figure 2**. The CMR (P = 0.001) and YLL rate (P = 0.007) for deaths from ovarian cancer showed significantly increasing trends, while the trend of ASMRW remained relatively stable (P = 0.064). The CMR increased with an APCC of 2.92% (95% CI, 1.36% to 4.51%; P = 0.001, **Figure 2A**) per year. The YLL rate increased with an APCC of 2.02% (95% CI, 0.65% to 3.41%; P = 0.007, **Figure 2B**) during the study period. In terms of age-specific trends, CMR and YLL rate of the total population are shown in **Figures 2C, D**. Increasing CMR trends were seen in the age groups 60-69 (P = 0.045), and 70-79 (P = 0.017) years. YLL rates only increased in the age groups 70-79 years (P = 0.017), but did not significantly change in all other age groups.

### Quantitative impact of demographic and non-demographic factors on the CMR increase

**Figure 3:**
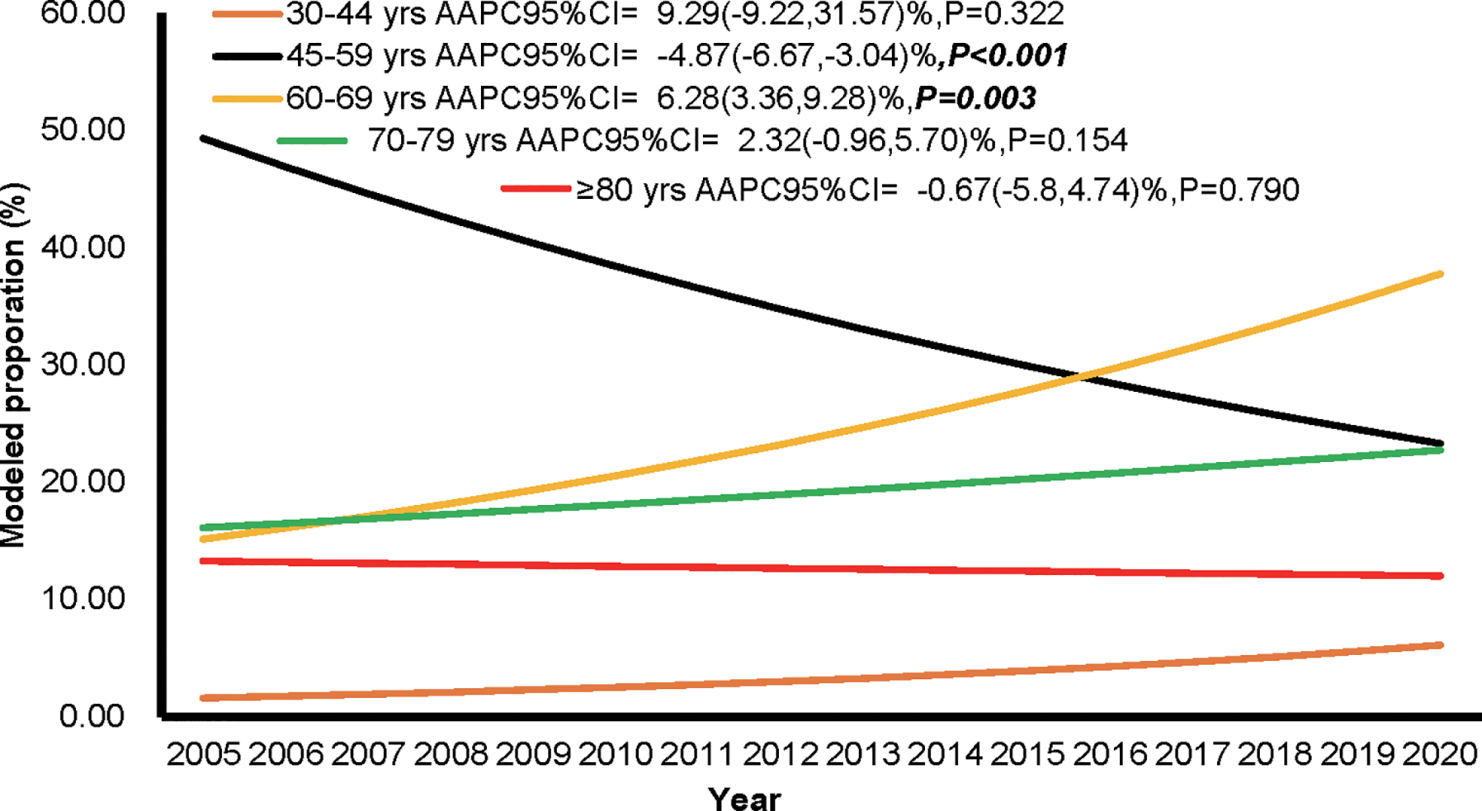
The proportion of individuals among different age groups during the period of 2005 to 2020 in Pudong New Area, Shanghai, China.

**Figure 4:**
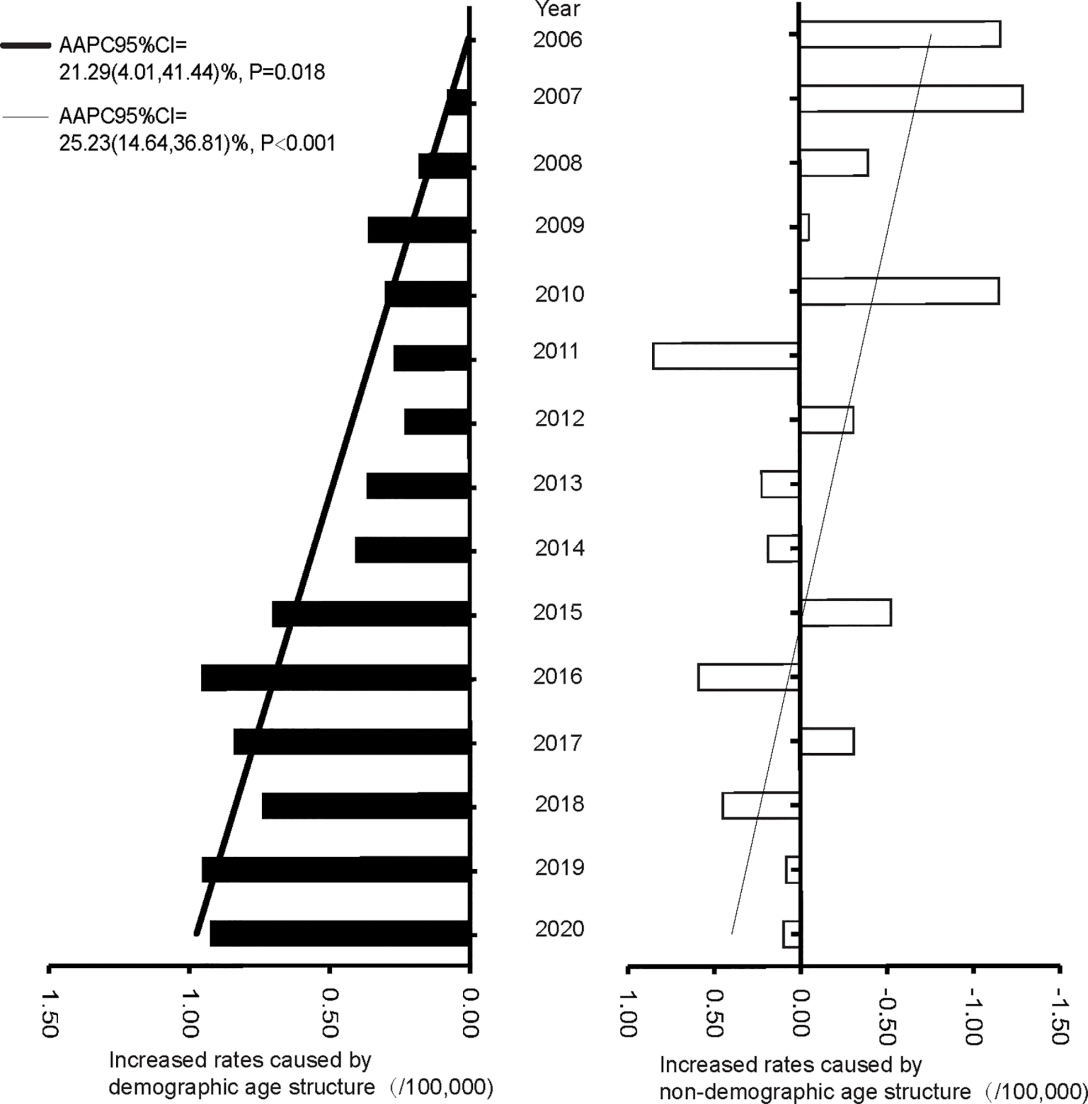
The increased rates caused by demographic and non-demographic factors from 2005 to 2020 in Pudong New Area, Shanghai, China.

The proportions of individuals aged 60-69 years showed an upward trend (AAPC of 6.28%, 95%CI: 3.36% to 9.28%, P = 0.003), while the age group 45-59 showed a downward trend (AAPC of -4.87%, 95%CI: -6.67% to -3.04%, P < 0.001) from 2005 to 2020. The age groups 45-59, 60-69, and 70-79 years were the top three in terms of population proportion (**Figure 3**). The trends of CMR increase caused by non-demographic and demographic factors are shown in **Figure 4** and **Supplementary Tables S5**. In the total population, the rates caused by non-demographic and demographic factors increased by 21.29% (95%CI: 4.01% to 41.44%, P = 0.018) and 25.23% (95%CI: 14.64% to 36.81%, P < 0.001), respectively.

### Trends of overall survival of ovarian cancer patients

**Figure 5:**
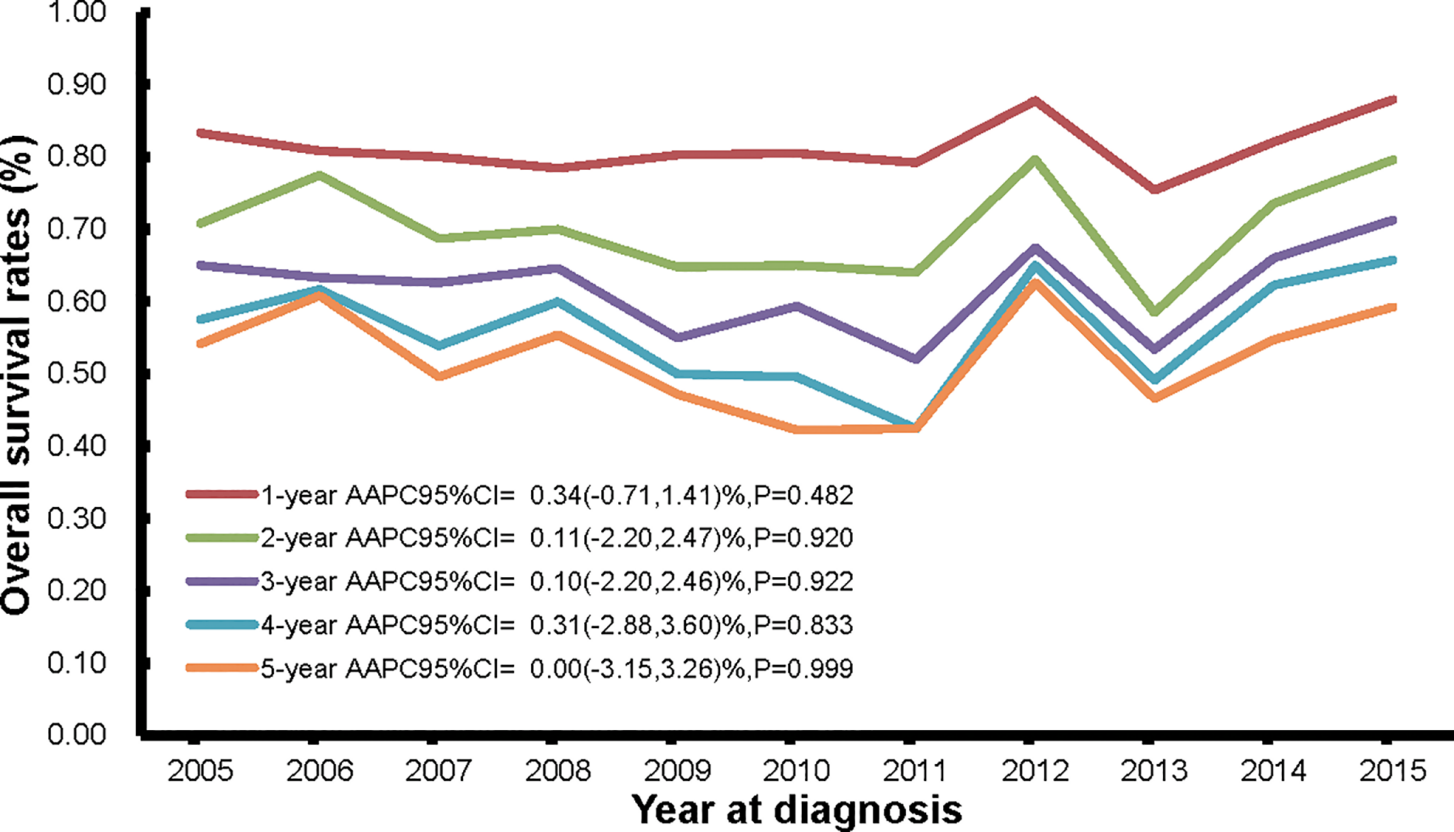
The trends in 1-year, 2-year, 3-year, 4-year, and 5-year overall survival rates of persons with ovarian cancer death from Pudong New Area, Shanghai, China, 2005-2015.

The current analysis included incident cases (n = 1033) of ovarian cancer from 2002 to 2015, with at least 5 years of follow-up time. Of the 1330 incident cases from year 2002 to 2015, 898 deaths (898/1330, 67.52%) occurred in the mortality database from year 2005-2020 (**Figure 1**), which not only supplemented and verified the incidence database, but also is the basis of our survival analysis. The temporal trends of 1-year to 5-year overall survival rates are shown in **Figure 5** and **Supplementary Tables S6**. The 1-, 2-, 3-, 4-, and 5-year overall survival rates were around 80% (range: 75.4%-88.0%), 70% (range: 58.5%-79.7%), 62% (range: 52.0%-71.3%), 56% (range: 42.4%-65.7%), and 50% (range: 42.3%-62.6%), respectively. In general, the overall survival rates stayed stable from 2005 to 2015.

## Discussion

In China, there were estimated 52,414 new cases of and 22,500 deaths caused by ovarian cancer in 2015, making it the second leading cause of cancer death of the female reproductive system (15). Due to lack of screening methods and less peculiar clinical symptoms, most of the patients are diagnosed at an advanced stage, leading to a poor prognosis (16). In the current analysis, based on all cause of death data, we analyzed underlying cause of ovarian cancer death. More importantly, we also provided data regarding multiple primary tumors (ovarian cancer and other cancer types) as the underlying cause of death. Often, statistics about underlying cause of ovarian cancer death obtained from death registries based on the cancer registration reporting system tend to count multiple primary tumors separately. Studies using ovarian cancer specific death data may under-estimate the actual burden of the disease. Our study results, therefore, to the greatest extent, have reflected the real situation of ovarian cancer related death among PNA residents.

Ovarian cancer is an aging-associated disease and more often occur in patients above 50 years (17). Our data showed that patients less than 45 years of age only accounted for 1.37% ovarian cancer related deaths (0-14 years: 0; 15-29 years: 0.31%; 30-44 years: 1.06%). Premature death from ovarian cancer in PNA is mainly among the age group of 60-79 years. Data from the Global Burden of Disease 2019 (18) have shown that in general World Bank low income countries have lower age-standardized mortality rate and YLL rate than high income countries (**Supplementary Tables S7**). In particular, PNA have similar age-standardized mortality rate and YLL rate compared to the Chinese national statistic (not including Taiwan province), but was lower than the global average from year 2005 to 2019 (**Supplementary Tables S7**). Further investigation of the data revealed that the PNA statistics are slightly lower than those of the India and Japan, and much lower than those of the United States during the same time period (**Supplementary Tables S7**). Factors including overweight and obesity, dietary patterns, and physical activities may explain the above regional differences (19, 20).

We observed a significant upward trend of CMR from 2005 to 2020. Based on the age structure of each year, the impact of population structure change on mortality rates was evaluated using the decomposition analysis. Our results showed that the high mortality rate of ovarian cancer is mainly attributed to the aging population. In addition, the significant upward trend of CMR is also the result of population aging, which is reflected by the stable age standardized rates. China is facing the challenge of population ageing. PNA has entered the ageing society in 2008 and super-ageing society in 2018, with the proportion of population aged over 65 years of 14.21% and 20.81%, respectively (11). Ovarian cancer is and will continue to be a major threat to women’s health in China in the foreseeable future.

Ovarian cancer is an aggressive disease, with a mortality-to-incidence ratio > 0.6 (2). To the best of our knowledge, our study is first of the kind that analyzed co-morbidity data of all causes of ovarian cancer death. Our data supported the notion that ovarian cancer is a highly fatal disease, and patients often died of multiple diseases. Based on the underlying cause of death data, the main co-morbidities in ovarian cancer death in PNA were diseases of the respiratory system, diseases of the digestive systems, and diseases of the circulatory system; and the main metastatic sites of ovarian cancer deaths were metastatic ovarian cancer to the liver, lung, retroperitoneum and peritoneum. Comprehensive management of ovarian cancer should take a holistic approach and improve patients’ overall life quality (21).

Despite advances in surgery and treatment strategies such as targeted therapy and immunotherapy brought by cutting-edge research, the survival rates of ovarian cancer have only improved modestly (2). Consistent with data from the United States and Canada (2), our analysis showed that the 5-year survival rate of ovarian cancer patients has stayed stable (around 50%) from 2005 to 2015 in PNA. The PNA Cancer Registry System and the PNA Mortality Registry System are two parallel and complementary registry systems, and analyzing data from both systems are more reliable than results obtained from a single system. Ovarian cancer is a heterogeneous disease and its aetiology remains elusive. In addition, different subtypes have distinct biological and molecular characteristics (2, 22). Technologies such as the next-generation sequencing and identification of promising new biomarkers may enable individualized precision medicine in ovarian cancer management, and finally improve survival (23, 24).

Prevention is also crucial in improving the survival of ovarian cancer patients. The development of ovarian cancer is a complex interplay of many risk factors, including genetic factors, epigenetic modifications, environmental factors, and lifestyle factors (17). Studies have suggested that lifestyle factors including alcohol consumption, obesity, cigarette smoking, unhealthy diet pattern, and physical inactivity are associated with increased ovarian cancer risk, although some are subtype specific (19, 20). Besides un-modifiable factors such as age, ethnicity, and genetics, some modifiable factors (e.g. healthy diet with enough leafy vegetables, allium vegetables and green tea intake, and increased physical activity) can affect ovarian cancer risk and survival (20, 23). It is well-established that use of oral contraceptive pill (OCP), pregnancy, breastfeeding, and tubal ligation are protective factors of ovarian cancer; other factors including use of aspirin and non-aspirin non-steroidal anti-inflammatory drugs may also reduce the risk of ovarian cancer (25, 26). For the moderate-to-high risk population (people with genetic predispositions), risk-reduction surgery and chemoprevention (such as OCPs and aspirin) can be beneficial (25, 27). Therefore, it is important to perform risk stratification and further personalize individual risk, so targeted prevention and management strategies can be implemented to reduce the life loss and relieve overall ovarian cancer disease burden.

Shanghai is the forerunner of economic development and modernization in China, and has become one of the earliest cities entering the upper-middle-income region in China. Alongside the rapid urbanization, Shanghai is also the first city entering the ageing society in China (9). PNA, founded in 1993, is the largest district of Shanghai (28). PNA has a permanent resident population of 3.22 million, accounting for approximately 20% of the total permanent resident population in Shanghai in 2018 (28). PNA represents the areas undergoing rapid modernization and is considered an ideal sample to study the effect of demographic and socioeconomic changes on disease burden. Moreover, PNA has a reliable mortality registration system covering 100% of the permanent resident population with periodical evaluations and compilations following standard guidelines, which ensures the data quality and completeness (9).

The current study has several limitations. First, all our data were from the PNA. Although it is the largest district in Shanghai, the study results need to be interpreted with caution and the generalizability needs to be further validated. Second, we had data on major co-morbidities, but not on patients’ lifestyle, treatment, surgery, all factors closely related to the occurrence and severity of ovarian cancer. Therefore, it is hard to study the contribution of all factors in ovarian cancer related mortality. Nonetheless, data used in this study was from the government monitoring system in the developed region of a country experiencing rapid transition, which ensured data quality and reliability. As it is difficult to obtain complete long-term data in less developed regions due to imperfect information system, such studies are hard to perform.

## Conclusions

In conclusion, this study reports the longitudinal changes of ovarian cancer death patterns in Shanghai from 2005 to 2020. The disease burden of ovarian cancer patients in a transition period with drastic economic and social transformation were analyzed. With an ageing population, the increasing health awareness and pursuit of life quality, ovarian cancer is and will remain to be a significant health issue for Chinese women. Therefore, it is important to identify cost-effective modifiable factors, and developing corresponding strategies to reduce the disease burden. What is more, continued efforts are needed in improving the early detection and comprehensive management of ovarian cancer, so as to finally reduce ovarian cancer related mortality in this population. Finally, our study results have provided valuable information and reference to other cities and regions undergoing similar transitions (urbanization and ageing), in terms of how to balance population health and economic growth, and to achieve high-quality development.

## Data availability statement

The data analyzed in this study is subject to the following licenses/restrictions: The data that support the findings of this study are available from the Centre for Disease Control and Prevention of the Pudong New Area, Shanghai, but restrictions apply to the availability of the data which were used under licence for the current study; therefore, they are not publicly available. However, data are available from the authors upon reasonable request and with permission from the Centre for Disease Control and Prevention of the Pudong New Area. Requests to access these datasets should be directed to XL: xiaopanli0224@126.com.

## Ethics statement

The study protocol followed the 2000 Declaration of Helsinki and was approved by the Ethics Committee of the Shanghai PNA Centre for Disease Control and Prevention (No. 2016-04-0586). Written informed consent to participate in this study was provided by the participants’ legal guardian/next of kin.

## Author contributions

Conceptualization: XL and YD. Data curation: XL and YC. Formal analysis: XL, MZ, and HL. Funding acquisition: YC, HL, and YD. Supervision: HL and YD. Writing – original draft: MZ and YD. Writing – review & editing: all authors. All authors contributed to the article and approved the submitted version.

## Funding

This study was supported by the National Natural Science Foundation of China (No. 81973119 to YD and No. 81904014 to HL) and the Shanghai Public Health System Construction Three-year Action Plan Outstanding Youth Talent Training Program (No. GWV-10.2-YQ43 to YC).

## Acknowledgments

The authors thank all staff in the vital statistics system of the Pudong New Area for their great work in data collection and assuring data quality.

## Conflict of interest

The authors declare that the research was conducted in the absence of any commercial or financial relationships that could be construed as a potential conflict of interest.

## Publisher’s note

All claims expressed in this article are solely those of the authors and do not necessarily represent those of their affiliated organizations, or those of the publisher, the editors and the reviewers. Any product that may be evaluated in this article, or claim that may be made by its manufacturer, is not guaranteed or endorsed by the publisher.

## References

[1] Sung H Ferlay J Siegel RL Laversanne M Soerjomataram I Jemal A. Global cancer statistics 2020: GLOBOCAN estimates of incidence and mortality worldwide for 36 cancers in 185 countries. CA Cancer J Clin (2021) 71(3):209–49. doi: 10.3322/caac.21660 33538338

[2] Lheureux S Braunstein M Oza AM. Epithelial ovarian cancer: Evolution of management in the era of precision medicine. CA Cancer J Clin (2019) 69(4):280–304. doi: 10.3322/caac.21559 31099893

[3] Odunsi K. Immunotherapy in ovarian cancer. Ann Oncol (2017) 28(suppl_8):viii1–7. doi: 10.1093/annonc/mdx444 PMC5834124 29232467

[4] Gersekowski K Delahunty R Alsop K Goode EL Cunningham JM Winham SJ. Germline BRCA variants, lifestyle and ovarian cancer survival. Gynecol Oncol (2022) 165(3):437–45. doi: 10.1016/j.ygyno.2022.03.020 PMC9133192 35400525

[5] Yang C Xia BR Zhang ZC Zhang YJ Lou G Jin WL. Immunotherapy for ovarian cancer: Adjuvant, combination, and neoadjuvant. Front Immunol (2020) 11:577869. doi: 10.3389/fimmu.2020.577869 33123161 PMC7572849

[6] Herrmann C Ess S Thürlimann B Probst-Hensch N Vounatsou P. 40 years of progress in female cancer death risk: A Bayesian spatio-temporal mapping analysis in Switzerland. BMC Cancer (2015) 15:666. doi: 10.1186/s12885-015-1660-8 26453319 PMC4600311

[7] Majdan M Plancikova D Maas A Polinder S Feigin V Theadom A. Years of life lost due to traumatic brain injury in Europe: a cross-sectional analysis of 16 countries. PloS Med (2017) 14(7):e1002331. doi: 10.1371/journal.pmed.1002331 28700588 PMC5507416

[8] Luo Z He Y Ma G Deng Y Chen Y Zhou Y. Years of life lost due to premature death and their trends in people with malignant neoplasm of female genital organs in shanghai, China during 1995-2018: a population based study. BMC Public Health (2020) 20(1):1489. doi: 10.1186/s12889-020-09593-6 33004024 PMC7528500

[9] Luo Z Lv H Chen Y Xu X Liu K Li X. Years of life lost due to premature death and their trends in people with selected neurological disorders in shanghai, China, 1995-2018: A population-based study. Front Neurol (2021) 12:625042. doi: 10.3389/fneur.2021.625042 33746880 PMC7973274

[10] Chen H Hao L Yang C Yan B Sun Q Sun L. Understanding the rapid increase in life expectancy in shanghai, China: A population-based retrospective analysis. BMC Public Health (2018) 18(1):256. doi: 10.1186/s12889-018-5112-7 29444657 PMC5813363

[11] Shanghai Bureau of Statistics. 2020 shanghai statistical yearbook . Available at: http://tjj.sh.gov.cn/tjnj/nj20.htm?d1=2020tjnj/C0202.htm (Accessed 24 June 2022).

[12] Li X Zhou Y Luo Z Gu Y Chen Y Yang C. The impact of screening on the survival of colorectal cancer in shanghai, China: A population based study. BMC Public Health (2019) 19(1):1016. doi: 10.1186/s12889-019-7318-8 31357981 PMC6664771

[13] Paoin W Yuenyongsuwan M Yokobori Y Endo H Kim S. Development of the ICD-10 simplified version and field test. Health Inf Manage (2018) 47(2):77–84. doi: 10.1177/1833358317701277 28537209

[14] Cheng X Yang Y Schwebel DC Liu Z Li L Cheng P. Population ageing and mortality during 1990–2017: A global decomposition analysis. PloS Med (2020) 17(6):e1003138. doi: 10.1371/journal.pmed.1003138 32511229 PMC7279585

[15] Chen W Zheng R Baade PD Zhang S Zeng H Bray F. Cancer statistics in China, 2015. CA Cancer J Clin (2016) 66(2):115–32. doi: 10.3322/caac.21338 26808342

[16] Torre LA Trabert B DeSantis CE Miller KD Samimi G Runowicz CD. Ovarian cancer statistics, 2018. CA Cancer J Clin (2018) 68(4):284–96. doi: 10.3322/caac.21456 PMC6621554 29809280

[17] Roett MA Evans P. Ovarian cancer: An overview. Am Fam Physician (2009) 80(6):609–16. Available at: https://www.aafp.org/pubs/afp/issues/2009/0915/p609.html 19817326

[18] Global burden of disease study 2019 (GBD 2019) results (2020). Available at: http://ghdx.healthdata.org/gbd-results-tool.

[19] Grosso G Bella F Godos J Sciacca S Del Rio D Ray S. Possible role of diet in cancer: Systematic review and multiple meta-analyses of dietary patterns, lifestyle factors, and cancer risk. Nutr Rev (2017) 75(6):405–19. doi: 10.1093/nutrit/nux012 28969358

[20] Khodavandi A Alizadeh F Razis AFA. Association between dietary intake and risk of ovarian cancer: A systematic review and meta-analysis. Eur J Nutr (2021) 60(4):1707–36. doi: 10.1007/s00394-020-02332-y 32661683

[21] Wenzel L Osann K McKinney C Cella D Fulci G Scroggins MJ. Quality of life and adverse events: Prognostic relationships in long-term ovarian cancer survival. J Natl Cancer Inst (2021) 113(10):1369–78. doi: 10.1093/jnci/djab034 PMC8486331 33729494

[22] Peres LC Cushing-Haugen KL Köbel M Harris HR Berchuck A Rossing MA. Invasive epithelial ovarian cancer survival by histotype and disease stage. J Natl Cancer Inst (2019) 111(1):60–8. doi: 10.1093/jnci/djy071 PMC6335112 29718305

[23] Considine DPC Jia G Shu X Schildkraut JM Pharoah PDP Zheng W. Genetically predicted circulating protein biomarkers and ovarian cancer risk. Gynecol Oncol (2021) 160(2):506–13. doi: 10.1016/j.ygyno.2020.11.016 PMC7855757 33246661

[24] Ramus SJ Song H Dicks E Tyrer JP Rosenthal AN Intermaggio MP. Germline mutations in the BRIP1, BARD1, PALB2, and NBN genes in women with ovarian cancer. J Natl Cancer Inst (2015) 107(11):djv214. doi: 10.1093/jnci/djv21424 26315354 PMC4643629

[25] Menon U Karpinskyj C Gentry-Maharaj A. Ovarian cancer prevention and screening. Obstet Gynecol (2018) 131(5):909–27. doi: 10.1097/AOG.0000000000002580 29630008

[26] Doherty JA Jensen A Kelemen LE Pearce CL Poole E Schildkraut JM. Current gaps in ovarian cancer epidemiology: The need for new population-based research. J Natl Cancer Inst (2017) 109(10):djx144. doi: 10.1093/jnci/djx144 PMC6279295 29117355

[27] Xia YY Gronwald J Karlan B Lubinski J McCuaig JM Brooks J. Hereditary ovarian cancer clinical study group. Contraceptive use and the risk of ovarian cancer among women with a BRCA1 or BRCA2 mutation. Gynecol Oncol (2022) 164(3):514–21. doi: 10.1016/j.ygyno.2022.01.014 35063280

[28] Li X Qian M Zhao G Yang C Bao P Chen Y. The performance of a community-based colorectal cancer screening program: Evidence from shanghai pudong new area, China. Prev Med (2019) 118:243–50. doi: 10.1016/j.ypmed.2018.11.002 30412744

